# Proof-of-Concept Study of an Alpha-Fetoprotein-Derived Peptide for the Management of Canine Mammary Cancer

**DOI:** 10.3390/ani13030403

**Published:** 2023-01-25

**Authors:** James A. Bennett, Ann Hohenhaus, Thomas T. Andersen

**Affiliations:** 1 Department of Immunology and Microbial Disease, Albany Medical College, Albany, NY 12208, USA; 2 The Cancer Institute, Schwarzman Animal Medical Center, New York, NY 10065, USA; 3 Department of Molecular and Cellular Physiology, Albany Medical College, Albany, NY 12208, USA

**Keywords:** canine, mammary cancer, therapeutics, endogenous peptide analog, tolerability, prevention

## Abstract

**Simple Summary:**

Dogs needing treatment in addition to surgery for the management of mammary cancer should be given something that is not foreign to their system and that is well-tolerated and effective. Many canine mammary cancers are promoted in their growth by estrogen. Alpha-fetoprotein (AFP) is a mammalian protein that has anti-estrogenic properties. The anti-estrogenic site of AFP was isolated and developed into a drug referred to as AFPep. AFPep inhibited the development and growth of mammary cancers in rodents. The purpose of the study reported herein was to determine the tolerability of AFPep in normal and tumor-bearing dogs. Blood levels of AFPep, previously shown to inhibit mammary cancer growth in rodents, were achieved in dogs by injection as well as by the oral route of drug administration. In all cases, AFPep was well tolerated in dogs, as assessed by clinical behaviors as well as comprehensive blood tests. The data indicate that AFPep should be further developed for use against mammary cancer in dogs.

**Abstract:**

Novel, well-tolerated drugs are needed for the management of canine mammary cancer. Many of these cancers are promoted in their growth by estrogen. Alpha-fetoprotein (AFP) is a ubiquitous mammalian protein that has anti-estrogenic properties. AFPep (the anti-estrogenic site of AFP) has been developed into a readily synthesizable drug. AFPep has been shown to have anti-mammary cancer activity in several models of this disease, both in cell culture and in rodents. The purpose of the study reported herein was to determine the tolerability of AFPep in normal and tumor-bearing dogs. AFPep was given to dogs via both parenteral and oral routes in a single application and in repeated daily doses. Full clinical chemistry and hematology values were determined before and after drug administration. Blood levels of the drug were achieved in dogs that had been previously found to be oncostatic in rodents. No changes in clinical chemistry, hematology, and clinical behaviors were found in dogs following drug administration. The data support the further development of AFPep for clinical use against canine mammary cancer.

## 1. Introduction

Spontaneously occurring canine mammary gland tumors are a significant health concern, accounting for over 30% of canine cancers [1]. In dogs, mammary cancer development is, in part, regulated by reproductive hormone status [2,3,4]. In dogs undergoing ovariohysterectomy (OHE) prior to their first estrus, the incidence of mammary gland tumors is decreased compared to dogs spayed later in life [5]. In countries where OHE is routinely practiced, the rate of development of mammary gland tumors in dogs is lower than in countries where the procedure is uncommon [2,6]. Ovariohysterectomy at the time of mastectomy in sexually intact dogs has been associated with an improved prognosis [4,6,7]. Specifically, in dogs with a high level of serum 17β-estradiol, OHE conferred a protective effect [6]. The prolonged administration of progestins to female dogs increased the risk of mammary gland tumor development [8]. Approximately 50% of canine mammary cancers are estrogen-receptor-positive (ER+) [9].

Benign and malignant mammary gland tumors occur in dogs with approximately equal frequency [10,11]. Those dogs with benign tumors and some dogs with malignant tumors are cured by surgery. The current recommendation for the treatment of canine mammary gland tumors is the surgical excision of tumor tissue [4]. The extent of surgery (lumpectomy, simple, regional, or radical mastectomy) depends on the magnitude of surgery required for the complete removal of all tumor tissue. Complete excision can result in prolonged survival [4], whereas many dogs with less extensive interventions will develop additional tumors in the remaining mammary gland tissue [10]. Dogs with tumors defined as being highly malignant, based on histological grade or at an advanced clinical stage, develop metastatic disease [7]. Intuitively, chemotherapy seems to be an appropriate treatment for canine mammary gland tumors, although optimal chemotherapy protocols have not yet been defined [12,13]. Clearly, improved methods of treatment and research into the prevention of canine mammary gland tumors are needed.

In women, high levels of a naturally occurring, anti-estrogenic protein, alpha-fetoprotein (AFP), are protective against the development of breast cancer [14]. Extensive epidemiological studies have linked endogenous AFP to a reduced incidence of breast cancer [15,16,17]. AFP is produced by the fetus in utero and explains, in part, why parous women have a decreased risk of breast cancer [15]. AFP exhibits anti-breast cancer activity in multiple experimental studies [18,19,20,21,22,23]. This pre-existing evidence was a compelling and unique rationale for the development of AFPep (a peptide mimicking the anti-estrogenic effect of AFP [24,25]).

AFPep is a first-in-class medication that is useful for the treatment and prevention of breast cancer [26,27]. AFPep is a nine-amino acid, cyclic peptide derivative of a naturally occurring protein and is the anti-estrogenic, anti-breast-cancer active site of AFP [25]. None of the other active sites associated with AFP are contained in the peptide [28]. AFPep is well tolerated [28,29] and has a unique mechanism of action [30]. In mice and rats, AFPep is an effective additive in combination with tamoxifen for the prevention and treatment of breast cancer, and actually mitigates some of the toxicity (uterine hypertrophy) of tamoxifen [31]. AFPep is a multi-kinase inhibitor that culminates in the blockage of ER_α_ phosphorylation (activation) [30]. The potential utility of AFPep for the treatment and prevention of canine mammary cancer has not been thoroughly considered. Currently, there is a dearth of agents that can be administered to dogs to inhibit the growth of these estrogen-dependent tumors. 

Multiple studies into AFPep have been carried out against experimental mammary cancers growing in culture [30,32,33], and also as xenografts in rodents [24,26]. In addition, AFPep has been shown to inhibit the growth of primary cultures of estrogen receptor-positive cancer cells isolated from primary canine mammary tumors [9]. Therefore, it seems opportune to investigate the pharmacological properties of AFPep for the treatment and prevention of canine mammary cancer. The primary objective of this effort is to assess the tolerability of AFPep in higher mammalian species. Using mouse, dog, and monkey models, we report favorable pharmacokinetic and tolerability parameters for AFPep across these species, using doses and schedules that are therapeutic and preventive against mammary cancers in rodents.

## 2. Materials and Methods

### 2.1. AFPep

AFPep (sequence *cyclo*(EKTOVNOGN), where O is hydroxyproline [25,27]) was purchased from AmbioPharm Inc. (Augusta SC) in its lyophilized form and was confirmed to be 98% pure. Aliquots of lyophilized AFPep were solubilized in normal saline immediately before its use.

### 2.2. Animals

Mice were used for the pharmacokinetic and tolerability studies. ICR-SCID female mice that were 6–7 weeks old were obtained from Taconic Biosciences (Germantown, NY, USA). The mice were housed in micro-isolator cages, fitted with stainless steel wire lids and air filters. Cages were supported on ventilated racks supplying a HEPA-filtered air exchange.

### 2.3. Rats

Rats were used for tolerability and prevention studies. Two strains of rats were used in two different but well-established models of mammary cancer prevention. Sprague Dawley rats were used in studies of chemically induced mammary cancer, while ACI rats were used in studies of hormone-induced cancer. Female Sprague Dawley rats were obtained from Taconic Biosciences at 34 days of age and were placed on a controlled diet (Agway Pro-Lab 2000; Agway Corp, Syracuse, NY, USA) and allowed free access to food and water. August Copenhagen Irish (ACI) female rats were obtained from Envigo, Inc. (Indianapolis, IN, USA).

### 2.4. Dogs 

Dogs were used for pharmacokinetic and tolerability studies in Albany, while several breeds of companion dogs with spontaneously arising mammary cancer were assessed for AFPep tolerability and pharmacokinetics, using repeat-dose studies, at the Schwarzman Animal Medical Center in New York City. Sexually mature female beagle dogs were obtained from Marshall Farms, North Rose, NY, USA, and were singly housed in large indoor pens.

### 2.5. Monkeys 

Monkeys (*Macaca mulatta*, males) were contractually available through the Animal Resource Facility at Albany Medical College. The monkeys were used to assess pharmacokinetics and tolerability, in anticipation of future human studies. For dogs and monkeys, the full panels of clinical chemistry and hematology parameters were assessed. Monkeys were housed and cared for in accordance with the Guide for the Care and Use of Laboratory Animals (National Research Council, Washington, DC, USA). They were fed a standard Old World monkey diet, enriched with fruits and vegetables and other dietary novelties.

### 2.6. Mammary Cancer Xenografts 

Human MCF-7 breast cancer cells were adapted for growth in SCID mice with subcutaneous estrogen implants, as previously described [26]. Briefly, five tumor pieces (each 1.5 mm in diameter) were loaded into a 16-gauge trocar and deposited into the thoracic mammary fat pad. Tumors became palpable after approximately 21 days. Thereafter, tumor size was measured once daily with a Vernier caliper. Mice were randomized into control (saline) or treatment (AFPep 100 μg/mouse) groups and were treated via oral gavage administration of AFPep once daily for 14 days. Tumor volumes were calculated using the formula v = 0.52 (d)^2^D, assuming the tumor shape to be an ellipsoid of revolution around its long axis, D.

### 2.7. Methyl Nitroso Urea (MNU)-Induced Mammary Cancer Studies 

Fifty-day-old Sprague Dawley rats received a single injection of MNU (50 mg/kg) into the jugular vein [27]. Beginning 10 days after MNU, treatment with AFPep (4 mg/kg, s.c.) was initiated and continued once daily for 23 days. Rats in both the treated and control groups were palpated twice weekly for the detection of mammary tumors, noting the number, size, and location. Tumors were measured with calipers and the volume was calculated as described above. All animals were monitored daily for signs of toxicity.

### 2.8. Estrogen-Induced Mammary Cancers

ACI rats that weighed more than 100g were fitted subcutaneously with a 16-millimeter Silastic tubing implant containing estradiol [29]. AFPep treatment was given s.c. once daily. Rats were monitored for tumor development, as described above.

### 2.9. Pharmacokinetic (PK) Studies

AFPep (4 mg/kg) was administered by parenteral (i.v., s.c.) as well as oral routes to normal adult mice, dogs, and monkeys. Blood samples were obtained from anesthetized mice via the retroorbital plexus [26]. Blood samples from dogs and monkeys were obtained through an indwelling catheter in the cephalic vein by the veterinarian staff at Albany Medical College. Dogs were awake and alert during sampling; monkeys were under anesthesia, as previously described [26,34]. For the oral route, gavage was used in mice and monkeys; enteric capsules (DRcaps) containing AFPep were used and were readily swallowed by dogs. AFPep blood levels were measured using lliquid chromatography and mass spectrometry, as previously described [34].

The animals described above were housed in facilities certified by the American Association for the Accreditation of Laboratory Animal Care. The animal studies were carried out in adherence to the guidelines established in the guide for the care and use of laboratory animals with the approval of the Albany Medical College Animal Care and Use Committee.

### 2.10. Repeat-Dose Study of AFPep in Tumor-Bearing Dogs

Previous studies utilizing repeat dosing in mice [24] and rats [27] established safe starting doses for studies using dogs. Female dogs with one or more spontaneously occurring mammary gland tumors greater than approximately 2 cm in maximum diameter, presenting to the Cancer Institute at the Schwarzman Animal Medical Center in New York City, were screened for inclusion in the study. Dogs were included if they had a life expectancy of more than three months, no major organ dysfunction, or metastatic disease (as determined by three-view thoracic radiography and abdominal ultrasonography), precluding general anesthesia for biopsy, mastectomy, and OHE if not previously performed. All dogs underwent complete clinical staging, consisting of a complete blood count with cell differential, clinical chemistry, and urinalysis. Dogs were administered AFPep 10 mg s.c. once daily for 8 consecutive days. Tumors were biopsied prior to the administration of AFPep and were surgically removed on day 8 after the last injection of AFPep. Blood levels of AFPep were measured before and 30 minutes following its administration on the first day and eighth day of treatment with AFPep. Histopathology was obtained on all mammary gland tumors. Tolerability was assessed using the Veterinary Cooperative Oncology Group Common Terminology Criteria for Adverse Events [35]. Blood chemistry and hematology analyses were performed at IDEXX Laboratories Inc. (Westbrook, ME, USA), using the Sysmex XT and Beckman AU 5800 systems. This study was approved by the Schwarzman Animal Medical Center IACUC and informed consent was obtained in writing from all owners.

### 2.11. Data Analysis

For prevention studies, Fisher’s exact test was used to compare the number of animals with tumors; the log-rank test was used to compare the tumor incidence data [27]. A comparison of AFPep-treated animals with untreated animals was performed with an χ^2^ test. In the case of AFPep’s effect on tumor growth, the difference in tumor volumes between the treated and control groups was assessed using Student’s *t*-test. A minimum of 5 replicate animals were included per group. In this study, *p* < 0.05 is specified as significant. In Table 3, where there were only two animals in the groups that were pre- and post-AFPep, the mean ±range is reported.

## 3. Results

### 3.1. Efficacy of AFPep in Rodent Models

The efficacy of AFPep in various models of mammary cancer has been shown in multiple reports [24,26,27]. A pertinent summary of those reports is shown in Table 1. As a model for therapeutic intervention, MCF-7 human breast cancer xenografts that were established and growing in immunodeficient mice were almost completely stopped in their growth by the once-daily oral administration of 4 mg/kg AFPep over a 14-day period. In other studies using this model, when the once-daily administration of AFPep was begun immediately following tumor implantation, tumor outgrowth was completely prevented over the 30-day observation period [24]. Dose-response studies indicated that the minimum blood level of AFPep needed for the stoppage of xenograft growth was C_max_ = 0.1 μg/mL and the area under the curve (AUC) = 6.5 min μg/mL [26].

As models for preventive interventions, two widely used mammary cancer induction models in rats were studied to assess the cancer prevention capabilities of AFPep. In both chemically induced and hormone-induced mammary cancer models, AFPep reduced cancer incidence by almost 50% (see Table 1). The latency period of cancer appearance was also prolonged in both of these models [27,28,29]. 

### 3.2. Tolerability of AFPep in Rodent Models

During the AFPep treatment intervals, all appearance indicators such as body weight gain, cage activity, body temperature, gait, alertness, grooming, respiratory rate, and stool consistency in AFPep-treated mice were similar to that of the control mice, indicating that AFPep was being well-tolerated over that 30-day interval [24,26,28]. AFPep was administered once daily in rat studies, in some groups at very high doses (1000 ug/rat/day) over a 200-day interval. In no case was any toxicity seen in terms of animal behavior in the AFPep-treated groups, and upon necropsy, all organs appeared similar to those in the control groups [29].

### 3.3. Pharmacokinetics in Three Species

Tolerability, as well as pharmacokinetics studies, were extended into higher mammals, dogs, and monkeys. A 4 mg/kg dose was assessed parenterally, and the dosage was escalated to 100 mg/kg, administered orally. As shown in Table 2, the PK parameters were quite variable across rodent, canine, and primate species, which was not unexpected considering the differences in absorption and elimination across species. However, what was quite encouraging was that the C_max_ and AUC values were well above the minimum values (C_max_ 0.1 ug/mL, AUC 6.5 ug/mL) needed for efficacy in dose-response studies against human breast cancer xenografts growing in immune-deficient mice [26]. This provided further substantiation of the excellent tolerability of AFPep, as well as its applicability for oral use.

### 3.4. Tolerability in Higher Mammals

The clinical chemistry and hematology of this 4 mg/kg dose, given i.v. to normal beagle dogs and rhesus macaque monkeys, are shown in Table 3 and Table 4. In no case did exposure to AFPep result in significant changes to the clinical chemistry and hematology values in the studied animals. In monkeys, there were elevations in liver enzymes, but this was largely due to anesthesia [36] and the restraining of those animals needed for sampling. No such elevations in liver enzymes were seen in dogs, where no anesthesia and only mild manual restraint were necessary for blood sampling. All other clinical indicators in dogs, such as mobility, playfulness, appetite, stool consistency, heart rate, respiration rate, capillary refill time, and body temperature remained normal during these studies (Table 5). There was no evidence of reflux or vomiting after the administration of AFPep.

### 3.5. Repeat-Dosing Study in Dogs with Spontaneous Mammary Gland Tumors

An 8-day repeat dose study of AFPep was carried out in 6 dogs presenting with mammary cancer at the Schwarzman Animal Medical Center in New York City [37]. All dogs received 10 mg of AFPep subcutaneously once a day for 8 days. Four owners were instructed on how to administer the injection and prefilled syringes were dispensed for home administration. Empty syringes were returned as documentation regarding AFPep administration. Two dogs returned daily to the Schwarzman Animal Medical Center for the daily administration of AFPep. Blood samples were obtained pre- and 30 minutes post-AFPep administration for the measurement of AFPep blood levels. As shown in Table 6, AFPep blood levels were 2 to 3 ug/mL, which is well above the 0.1 ug/mL blood level shown to be efficacious in mammary tumor-bearing mice [26]. Complete blood counts with cell differential, clinical chemistry, and urinalysis, taken 30 minutes before the first exposure to AFPep and 30 minutes after the 8-day AFPep administration, were unchanged, indicating that the hematological, renal, and hepatic systems were operating within the normal range before and after the repeat dose of AFPep (Table 7). The only AFPep-related adverse event observed in this study was grade-1 injection site reactions in three out of six dogs. The six dogs in the repeat-dose study were all alive and well at a one-year post-tumor resection follow-up.

## 4. Discussion

The results of this study demonstrate that AFPep is well tolerated in dogs, even after repeated daily dosing, showing that AFPep is well tolerated in four separate species (mice, rats, dogs, and monkeys). The study has also shown that the oral administration route is a feasible option for the delivery of this drug in all of these species, sparing the discomfort from repeated needle sticks observed in 3 out of 6 dogs diagnosed with mammary gland cancer. Multiple AFPep exposures were carried out in normal dogs (beagles) to establish the pharmacokinetics of this drug in dogs. Repeated-dose exposures were carried out in diverse breeds of dogs presenting with mammary cancer, which further established the excellent tolerability of AFPep at blood levels that had previously been shown to be efficacious in rodent models of mammary cancer [26,31]. It has also been demonstrated that AFPep is well tolerated in primates after parenteral and oral administration, which bodes well for further clinical studies in companion animals and humans. The results of this study suggest that AFPep may be considered for adjuvant treatment of canine mammary cancer. Furthermore, it may be that AFPep could offer additional preventive efficacy in dogs that are at high risk of mammary cancer development.

New systemic agents are needed for the management of canine mammary cancer [12,13]. Post-operative chemotherapy protocols for dogs with high-grade or advanced clinical-stage mammary gland carcinoma have not been standardized and are accompanied by significant host toxicity. In extensive studies in rodents, and, now, preliminary studies in dogs, AFPep has not manifested any of the toxicities associated with cytotoxic chemotherapy and clearly impedes the growth of rodent mammary cancer and human breast cancer xenografts in vivo [24,27], and in canine and human breast cancer cells in culture [9].

In terms of new drug development, it should be emphasized that AFPep was derived from the natural mammalian protein, AFP. Therefore, it is probably not surprising that it has an excellent tolerability profile, especially considering that the fetal blood levels of AFP reach 10^−5^ M [38] and the blood levels of AFPep needed for anti-breast cancer activity are only 10^−7^ M [26]. During the development of AFPep, the full-length protein was parsed to locate the anti-estrogenic and anti-breast cancer site of AFP; then, that site was manipulated to achieve a readily synthesizable, stable, orally active cyclic peptide. Recent publications have shown the growing use of peptides for therapeutic purposes [39]. Peptides are advantageous in that they can have exquisite specificity for their intended receptor. When metabolized, peptides yield simple amino acids that are endogenous to normal mammalian physiology, which reduces the risk of conversion of the active drug to a toxic metabolite.

It is important to note that during the development of AFPep, head-to-tail cyclization [25] was carried out to increase stability as well as oral bioavailability. Other investigators have shown that the cyclization of peptides increases bioavailability [39,40,41]. In a recent study of over 12 anti-breast cancer cyclic peptides, AFPep was shown to have the most potency against the studied breast cancer cell lines [40]. Furthermore, Torres et al. [9,33] have shown that AFPep was effective against ER-positive primary canine mammary cancer cells growing in culture, suggesting that these types of cancers are likely to be susceptible to the anti-cancer effects of AFPep in vivo.

## 5. Conclusion

Following these demonstrations of chemical stability, bioavailability, tolerability, in vitro efficacy against cell lines, prevention capability, and in vivo efficacy against human breast-cancer xenografts growing in mice, it is appropriate to expand the use of this agent into efficacy studies against canine mammary cancers.

## Figures and Tables

**Table 1:** The therapeutic and preventive efficacy of AFPep * in rodent models of mammary cancer.

Treatment	MCF-7 Human Breast Cancer Growth in SCID Mice ^1^	MNU-Induced Mammary Tumors in Sprague Dawley Rats ^2^	Estrogen-Induced Mammary Tumors in ACI Rats ^3^
	Mm ^3^	% of Rats with Tumors	% of Rats with Tumors
Saline	750 + 20	78	72
AFPep	30 + 8 *	40 *	48 *

* AFPep is the anti-estrogenic site of the α-fetoprotein, *p* < 0.05; ^1^ Inhibition of human mammary tumor growth is a model for therapeutic use [24]. ^2^ Inhibition of chemically induced mammary cancer is a model for preventive use [27]. ^3^ Inhibition of estrogen-induced cancer appearance is a model for preventive use [28,29].

**Table 2:** Pharmacokinetic parameters for AFPep in mouse, dog, and primate models.

	i.v.			s.c.			p.o.		
	C_max_	AUC	T_1/2_	C_max_	AUC	T_1/2_	C_max_	AUC	T_1/2_
	µg/mL	min(µg/mL)	min	µg/mL	min(µg/mL)	min	µg/mL	min(µg/mL)	min
Mouse	12	252	11	6.4	207	11	0.14	4.8	6.3
Dog	35	900	18	5.8	812	65	0.10	14	39
Monkey	13	1574	107	8.2	1407	27	0.03	31	810

An effective dose in mice (100 µg/mouse, 4 mg/kg) was adjusted appropriately for dogs and primates and was administered via the routes indicated. Blood samples were collected at multiple time points and plasma was processed for the LC/MS/MS measurements of AFPep. Data were fitted via non-linear regression (Pharsight Phoenix 64 WinNonLin, Princeton NJ, USA) to hypothetical first-order PK models representing intravenous bolus dosages or extravascular dosages.

**Table 3:** Blood chemistry and hematology values before and 24 h after intravenous administration of 40 mg AFPep to dogs.

Parameter	Pre-AFPep	Post-AFPep
ALP	27 ± 4	33 ± 2
ALT	15 ± 1	16 ± 1
AST	28 ± 8	20 ± 1
Creatine Kinase	186 ± 20	135 ± 2.7
GGT	2 ± 1	3 ± 1
Albumin	3.3 ± 0.1	3.2 ± 0.1
Total Protein	5.9 ± 0.1	5.8 ± 0.1
Globulin	2.6 ± 2	2.6 ± 0.1
Total Bilirubin	0.1	0.1
Bilirubin Conjugated	0	0
BUN	14 ± 2	13.2 ± 2
Creatinine	0.6	0.7 ± 0.1
Cholesterol	176 ± 3	171 ± 2
Glucose	84 ± 3	93 ± 9
Calcium	10.1 ± 0.1	10.3 ± 0.4
Phosphorous	4.5 ± 0.1	4.6 ± 0.2
Bicarbonate T_CO2_	18 ± 1	21 ± 1
Chloride	115 ± 1	112 ± 1
Potassium	4.5 ± 0.1	4.7 ± 0.1
Sodium	147 ± 1	146 ± 2
WBC	6.6 ± 1	7.0 ± 3
RBC	6.7 ± 1	6.9 ± 1
HGB	15.8 ± 0.9	15.9 ± 1
HCT	45.3 ± 2	46 ± 1.5
MCV	67 ± 1	68 ± 0.8
MCH	23.4 ± 0.2	23.2 ± 1
Platelet	288 ± 12	274 ± 21

Average ± range from 2 dogs, each ~10 kg.

**Table 4:** Blood chemistry and hematology values before and 24 h after the intravenous administration of 40 mg AFPep to monkeys.

Parameter	Pre-AFPep	Post-AFPep
ALP	63 ± 12	66 ± 11
ALT	26 ± 2	**106 ± 40**
AST	20 ± 5	**146 ± 33**
Creatine Kinase	388 ± 52	**3769 ± 86**
GGT	48 ± 10	48 ± 12
Albumin	4.2 ± 0.1	4.0
Total Protein	6.9 ± 0.3	6.7 ± 0.3
Globulin	2.8 ± 0.3	2.7 ± 0.3
Total Bilirubin	0.1	0.1
Bilirubin Conjugated	0	0
BUN	15 ± 2	12 ± 2
Creatinine	1.0 ± 0.1	0.9 ± 0.1
Cholesterol	*104 ± 5*	*102 ± 9*
Glucose	60 ± 10	80 ± 5
Calcium	9.6 ± 0.1	8.8 ± 0.3
Phosphorous	4.2 ± 0.5	3.6 ± 0.6
Bicarbonate T_CO2_	24 ± 2	23 ± 2
Chloride	108 ± 1	*40 ± 1*
Potassium	5.1 ± 0.9	4.2 ± 0.7
Sodium	146 ± 1	145 ± 2
WBC	6.2 ± 0.2	7.3 ± 2.7
RBC	4.9 ± 0.6	4.6 ± 0.5
HGB	13.0 ± 1.1	12.0 ± 1.4
HCT	39 ± 6	36 ± 5
MCV	77 ± 2	78 ± 2
MCH	25.9 ± 0.3	25.7 ± 0.2
Platelet	273 ± 9	240 ± 2

Average ± SE from 6 monkeys, each ~10 kg. Values above the normal range are in bold. Values below the normal range are italicized.

**Table 5:** Clinical parameters in unanesthetized dogs.

	Pre AFPep	30 min Post AFPep
Heart Rate	114 ± 4	112 ± 5
Respiration Rate	27 ± 3	28 ± 4
Capillary refill time	<2 s	<2 s
Body temperature	38.9 °C	39.0 °C

Mean ± SE from 6 dogs.

**Table 6:** An 8-day repeat-dose study of AFPep blood levels in dogs (in ng/mL) ^a^.

Dog			Day 1		Day 8	
Breed	Age (years)	Weight (kg)	Pre-Treatment	Post-Treatment	Pre-Treatment	Post-Treatment
Pomeranian	11	7.98	1.2	2400	0	2100
Yorkshire terrier	9	5.86	0	2200	0	1900
Poodle mix	11	5.80	0	4400	8	5000
Pitbull	10	30.2	2.8	9000	0	1200
Shih tzu	8	11.02	0	800	0	1200
Chihuahua	5	7.53	0	2500	0	3300
Mean ± S.D.			0.7 ± 0.5	3600 ± 1200	1.3 ± 1.3	2500 ± 600

^a^ 10 mg of AFPep given s.c. once daily for 8 days.

**Table 7:** Blood chemistry and hematology values in an 8-day repeat-dose AFPep study in dogs ^a^.

	Dog 1		Dog 2		Dog 3		Dog 4		Dog 5		Dog 6	
	Pre ^b^	Post ^c^	Pre	Post	Pre	Post	Pre	Post	Pre	Post	Pre	Post
ALP	**360**	**261**	128	115	**589**	**178**	158	148	16	15	25	19
ALT	51	35	13	14	54	39	49	60	34	29	57	41
AST	35	14	37	20	24	15	24	23	36	26	25	18
Creatine Kinase	**297**	120	**408**	**225**	165	115	153	169	**288**	138	93	80
GGT	4	4	5	6	4	4	2	6	5	**18**	4	5
Albumin	3	3	4	4	3	4	3	3	4	4	3	3
Total Protein	7	6	8	8	6	7	6	6	7	7	7	6
Globulin	4	3	4	4	3	3	3	4	3	3	4	3
Total Bilirubin	0.1	0.1	0.1	0.1	0.1	0.1	0.1	0.1	0.1	0.1	0.1	0.1
Bilirubin Conj.	0.1	0.1	0.1	0.1	0.1	0.1	0.1	0.1	0.1	0.1	0.1	0.1
BUN	24	27	12	14	21	14	**36**	24	24	15	40	30
Creatine	0.9	0.8	0.6	0.8	0.7	0.6	0.9	1.0	0.6	0.7	0.9	0.7
Cholesterol	320	277	257		234	273	**458**	**451**	**390**	**373**	206	198
Glucose	102	107	84	178	95	107	98	92	99	88	80	81
Calcium	10	10	10	11	10	9	10	10	10	10	10	10
Phosphorous	4	4	4	3	4	4	4	3	2	3	5	6
Bicarbonate	**30**	21	22	23	23	23	22	17	21	21	22	21
Chloride	112	111	110	108	111	108	112	114	111	110	113	114
Potassium	5	4	5	5	5	5	4.6	4.4	4	5	4	4
Sodium	146	145	146	146	146	145	148	147	145	143	145	143
WBC	15	18	10	7	8	6	13	13	7	7	15	13
RBC	6	6	8	7	7	7	8	8	8	7	6	6
HGB	14	13	18	17	16	17	20	18	18	18	15	15
HCT	42	39	48	46	44	46	55	51	50	48	44	43
MCV	69	68	68	65	65	64	63	62	66	67	74	74
MCH	24	24	25	23	24	23	23	23	24	25	25	25
Platelets	**916**	438	453	**700**	**536**	**558**	**504**	**485**	**497**	**462**	**514**	**519**

Bold font indicates a parameter above the normal range. Italic font indicates a parameter below the normal range. ^a^ 10 mg of AFPep given s.c. once daily for 8 days. ^b^ Before the introduction of AFPep at the start of the study. ^c^ 30 min after the last injection of AFPep on day 8 of the study.

## Data Availability

The data presented in this study are available in this article.
